# Natural Products as New Treatment Options for Trichomoniasis: A Molecular Docking Investigation

**DOI:** 10.3390/scipharm85010005

**Published:** 2017-01-27

**Authors:** Mary Snow Setzer, Kendall G. Byler, Ifedayo Victor Ogungbe, William N. Setzer

**Affiliations:** 1 Department of Chemistry, University of Alabama in Huntsville, Huntsville, AL 35899, USA; mary.setzer@uah.edu (M.S.S.); kgb0011@uah.edu (K.G.B.); 2 Department of Chemistry & Biochemistry, Jackson State University, Jackson, MS 39217, USA; ifedayo.v.ogungbe@jsums.edu

**Keywords:** emerging infectious disease, *Trichomonas vaginalis*, in silico, drug discovery

## Abstract

Trichomoniasis, caused by the parasitic protozoan *Trichomonas vaginalis*, is the most common non-viral sexually-transmitted disease, and there can be severe complications from trichomoniasis. Antibiotic resistance in *T. vaginalis* is increasing, but there are currently no alternatives treatment options. There is a need to discover and develop new chemotherapeutic alternatives. Plant-derived natural products have long served as sources for new medicinal agents, as well as new leads for drug discovery and development. In this work, we have carried out an in silico screening of 952 antiprotozoal phytochemicals with specific protein drug targets of *T. vaginalis*. A total of 42 compounds showed remarkable docking properties to *T. vaginalis* methionine gamma-lyase (TvMGL) and to *T. vaginalis* purine nucleoside phosphorylase (TvPNP). The most promising ligands were polyphenolic compounds, and several of these showed docking properties superior to either co-crystallized ligands or synthetic enzyme inhibitors.

## 1. Introduction

Trichomoniasis is a sexually-transmitted disease (STD) caused by the parasitic protozoan *Trichomonas vaginalis* and is the most common non-viral STD with an estimated 3.7 million cases in the United States [1]. Only about 30% of individuals infected with *T. vaginalis* experience symptoms of genital discomfort, itching, burning or discharge, but there can be severe inflammation, increased risk of HIV infection, cervical cancer, preterm delivery and low birth weight [1]. Trichomoniasis can be treated with antibiotics, usually metronidazole or tinidazole, but there are increasing reports of resistance to these drugs [2]. There are currently no alternative drugs approved for the treatment of refractory cases of trichomoniasis, emphasizing the need for new treatment options. Recent investigations have identified several *T. vaginalis* proteins that may serve as targets for drug discovery and development [3,4]. 

### 1.1. *Trichomonas vaginalis* Protein Targets

Proteases are known to carry out important biological processes in parasitic protozoa and are therefore potential drug targets. Papain-like proteases have been suggested to be involved in nutrition and hemolysis, as well as able to induce apoptosis in human vaginal epithelial cells [3]. More than 40 papain-like cysteine proteases have been identified in the *T. vaginalis* degradome, which have been implicated as virulence factors [4].

Triosephosphate isomerase (TPI) is a glycolytic enzyme that catalyzes the interconversion of glyceraldehyde 3-phosphate and dihydroxyacetone phosphate and is an essential component of the glycolytic pathway [5]. Because of its importance in glycolysis, TPI has been identified as a good drug target for antiparasitic chemotherapeutics [6].

Lactate dehydrogenase (LDH) catalyzes the interconversion of lactate to pyruvate with concomitant interconversion of NAD^+^ to NADH. LDH is a key enzyme in glycolysis and is found in nearly all living cells. Because *T. vaginalis* lactate dehydrogenase (TvLDH) is required for parasite survival, but is not similar to human LDH, TvLDH may be regarded as a suitable target for drug discovery [7,8].

Methionine gamma-lyase (MGL) has been characterized in several bacteria species, as well as the parasitic protozoans *Entamoeba histolytica* and *Trichomonas vaginalis* [9]. The enzyme degrades sulfur-containing amino acids to α-keto acids, ammonia and thiols and plays a key role in the regulation of sulfur-containing amino acids. Mammals do not have MGL, so this enzyme is a potential drug target for anti-*Trichomonas* chemotherapy.

Thioredoxin reductase (TrxR) catalyzes the reduction of thioredoxin, and the thioredoxin system provides a defense against oxidative damage due to oxygen metabolism and redox signaling [10]. Mammalian TrxRs and TrxRs from parasitic protozoa are different classes with different mechanisms of activity [11]. Because TrxR is a strong antioxidant that protects *T. vaginalis* from oxidative stress, the parasite lacks glutathione or catalase and *T. vaginalis* thioredoxin reductase (TvTrxR) is very different from human thioredoxin reductase (HsTrxR), TvTrxR has been identified as a target for trichomoniasis chemotherapy [12] and is the target of metronidazole and other nitroimidazole drugs [13].

Purine nucleoside phosphorylase (PNP) catalyzes the phosphorolysis of the *N*-glycosidic bonds of purine nucleosides (or deoxynucleosides) to give α-ribose-1-phosphate and the purine base and functions in the purine salvage pathway [14]. Purine salvage is essential for obligate parasitic protozoa, including *T. vaginalis*, and *T. vaginalis* purine nucleoside phosphorylase (TvPNP) has been identified as an attractive chemotherapeutic target [15].

### 1.2. Homology Modeling

In the absence of an experimentally-determined protein structure by crystallographic or nuclear magnetic resonance (NMR) methods, homology modeling can provide useful three-dimensional structures for proteins that are related to known protein structures. The premise is that the proteins have similar structures and binding and/or active sites of the proteins retain identical structures. Several computational methods for predicting protein structures based on homology models are currently available [16], and homology modeling has been shown to be a valuable tool for in silico screening of biomolecular targets [17]. In this work, we have used homology modeling to predict *T. vaginalis* protein structures for which there are no experimentally-determined structures.

### 1.3. Molecular Docking

Molecular docking is a well-accepted tool in drug discovery and complements X-ray crystallography and NMR spectroscopy in analyzing small molecule-protein interactions. In many cases, it has replaced high-throughput screening for initial investigations in lead discovery. Nevertheless, there are some limitations to the method that mostly arise from not accounting for local and global protein dynamics, as well as the inability to accurately predict ligand-protein covalent interactions and solvent accessibilities. The protein is typically modeled as a rigid structure without flexibility; solvation in the active or binding site is usually not included in the models, and free-energies of the ligand-protein complexes are generally ignored [18,19,20]. In spite of these limitations, molecular docking studies of natural product ligands with potential drug targets can serve to identify natural product drugs or drug leads to treat human infections [6]. In this work, we have carried out in silico screening of antiprotozoal natural products with several potential protein targets of *Trichomonas vaginalis*.

## 2. Materials and Methods

### 2.1. Homology Modeling

Homology models for each of the *Trichomonas* proteins that are not currently available from the Protein Data Bank (PDB) were constructed from crystal structure templates found in the Protein Data Bank using FASTA sequences downloaded from the National Center for Biotechnology Information’s (NCBI) GenBank. Sequences with high sequence similarity in the PDB were identified with NCBI’s BLAST utility using the BLOSUM80 scoring matrix (BLOcks SUbstitution Matrix). Sequences with high similarity to the reference sequences, as well as having good coverage for the active sites in the proteins, were identified using NCBI’s BLAST utility with the BLOSUM80 scoring matrix and used as templates for single-reference homology modeling.

The protein sequences were first aligned to their respective template sequences using the BLOSUM62 substitution matrix and a protein backbone constructed and superposed to the reference structure using the protein alignment tool in Molecular Operating Environment, MOE 2014.0901. The homology modeling interface in MOE was used to generate a set of putative protein structures by aligning atomic coordinates of the amino acid sequence to those of the template sequence backbone and minimizing permutations of side chain orientations using the AMBER12:EHT force field [21,22,23] with reaction field solvation. The candidate structure with the lowest root-mean-square deviation of atomic positions (RMSD) deviation from the template backbone was selected and optimized using a constrained minimization.

The homology model for the *T. vaginalis* thioredoxin reductase (TvTrxR) sequence (Accession Number CAD47837.1) was generated from the closest matching template in the PDB, a thioredoxin reductase from *Brucella melitensis* (4JNQ [24], 69.1% sequence similarity, 81.5% site similarity, E = 2 × 10^−89^, RMSD = 1.27 Å, site RMSD = 0.94 Å). This structure was co-crystallized with a bound ligand, dihydroflavine-adenine dinucleotide, which was also used in the structure refinement of the final homology. The homology model for the *T. vaginalis* papain-like cysteine protease C2 (TvCP2) sequence (Accession Number AAR37420.1) was generated from the crystal structure of cathepsin K from *Oryctolagus cuniculus* (2F7D [25], 66.0% sequence similarity, 73.9% site similarity, E = 5 × 10^−71^, RMSD = 0.62 Å, site RMSD = 0.32 Å). This structure also has a bound nitrile inhibitor, (1*R*,2*R*)-*N*-(2-aminoethyl)-2-{[(4-methoxyphenyl)sulfonyl]methyl}cyclohexanecarboxamide, which was used in structure refinement of the final model. The homology model for *T. vaginalis* cathepsin L-like protease (TvCPCAC1) sequence (Accession Number EAY13782.1) was generated from the crystal structure of human cathepsin V (1FH0 [26], 61.5% sequence similarity, 75.0% site similarity, E = 6 × 10^−66^, RMSD = 0.76 Å, site RMSD = 0.60 Å) that was co-crystallized with a vinyl sulfone inhibitor, *N*-α-[(4-methylpiperazin-1-yl)carbonyl]-*N*-[(*3S*)-1-phenyl-5-(phenylsulfonyl)pentan-3-yl]- l-phenylalaninamide, which was included in the refinement of the final model. 

### 2.2. Molecular Docking

Protein-ligand docking studies were carried out based on the structures of potential *Trichomonas vaginalis* protein drug targets: *T. vaginalis* methionine gamma-lyase (TvMGL, PDB 1E5E and 1E5F [27]); *T. vaginalis* purine nucleoside phosphorylase (TvPNP, PDB 1Z34 and 1Z36 [28] and PDB 2ISC [29]); *T. vaginalis* triosephosphate isomerase (TvTPI, PDB 3QST [30] and PDB 4O4V [31]); *T. vaginalis* lactate dehydrogenase (TvLDH, PDB 4UUN and 5A1T [32]); *T. vaginalis* thioredoxin reductase (TvTrxR, homology model based on the crystal structure of *Brucella melitensis* TrxR, PDB 4JNQ [24]); *T. vaginalis* papain-like cysteine protease (TvCP2, homology model based on the crystal structure of rabbit cathepsin K, PDB 2F7D [25]); and *T. vaginalis* cathepsin L-like cysteine protease (TvCPCAC1, homology model based on the crystal structure of human cathepsin V, PDB 1FH0 [26]). In order to test for the selectivity toward *T. vaginalis* protein targets over human isozymes, molecular docking of the phytochemical ligands was also carried out on human PNP (HsPNP, PDB 3BGS [33] and 3INY [34]); human TPI (HsTPI, PDB 2JK2 [35] and 4POC [36]); human cathepsin K (HsCatK, PDB 1MEM [37] and 1U9V [38]); and human cathepsin L (HsCatL, PDB 3HWN [39] and 3OF8 [40]). 

Prior to docking, all solvent molecules and the co-crystallized ligands were removed from the structures. If co-factors were present, they were retained in each protein model (i.e., dihydroflavin adenine dinucleotide (FDA) in TvTrxR and 1,4-dihydronicotinamide adenine dinucleotide (NADH) in TvLDH). Molecular docking calculations for all compounds with each of the proteins were undertaken using Molegro Virtual Docker (Version 6.0.1, Molegro ApS, Aarhus, Denmark) [41], with a sphere (15 Å radius) large enough to accommodate the cavity centered on the binding sites of each protein structure in order to allow each ligand to search. If a co-crystallized inhibitor or substrate was present in the structure, then that site was chosen as the binding site. If no co-crystallized ligand was present, then suitably-sized (>25 Å^3^) cavities were used as potential binding sites. Standard protonation states of the proteins based on neutral pH were used in the docking studies. Each protein was used as a rigid model structure; no relaxation of the protein was performed. Assignments of the charges on each protein were based on standard templates as part of the Molegro Virtual Docker program; no other charges were necessary to set. Our in-house library of antiprotozoal phytochemicals (obtained by searching the phytochemical literature and the *Dictionary of Natural Products* [42]), which was filtered for drug-like properties based on Lipinski’s rule-of-five [43], was used for the molecular docking study. Overall, 952 antiprotozoal phytochemicals have been docked. This molecule set was comprised of 214 alkaloids, 369 terpenoids, 174 flavonoids and 195 polyphenolic compounds. Each ligand structure was built using Spartan‘16 for Windows (Version 1.1.0, Wavefunction Inc., Irvine, CA, USA). For each ligand, a conformational search and geometry optimization was carried out using the MMFF (Merck Molecular Force Field) [44]. Flexible ligand models were used in the docking and subsequent optimization scheme. Variable orientations of each of the ligands were searched and ranked based on their re-rank score. For each docking simulation, the maximum number of iterations for the docking algorithm was set to 1500, with a maximum population size of 50 and 100 runs per ligand. The RMSD threshold for multiple poses was set to 1.00 Å. The generated poses from each ligand were sorted by the calculated re-rank score. In analyzing the docking scores, we have attempted to account for the recognized bias toward high molecular weight compounds [45,46,47,48,49,50], using the scheme: DS_norm_ = 7.2 × E_dock_/MW^⅓^, where DS_norm_ is the normalized docking score, E_dock_ is the MolDock re-rank score, MW is the molecular weight and 7.2 is a scaling constant to bring the average DS_norm_ values comparable to E_dock_ [51].

As a test of docking accuracy and for docking energy comparison, co-crystallized ligands were re-docked into the protein structures (see Table 1). In addition, as a validation of the docking method, we have carried out docking of picomolar and nanomolar synthetic purine nucleoside phosphorylase inhibitors with human PNP and *T. vaginalis* PNP (see Table 2). The docking shows good docking scores and good docking pose orientations for these compounds serving to confirm the validity of the docking method.

## 3. Results and Discussion

### 3.1. Homology Modeling

**Figure 1:**
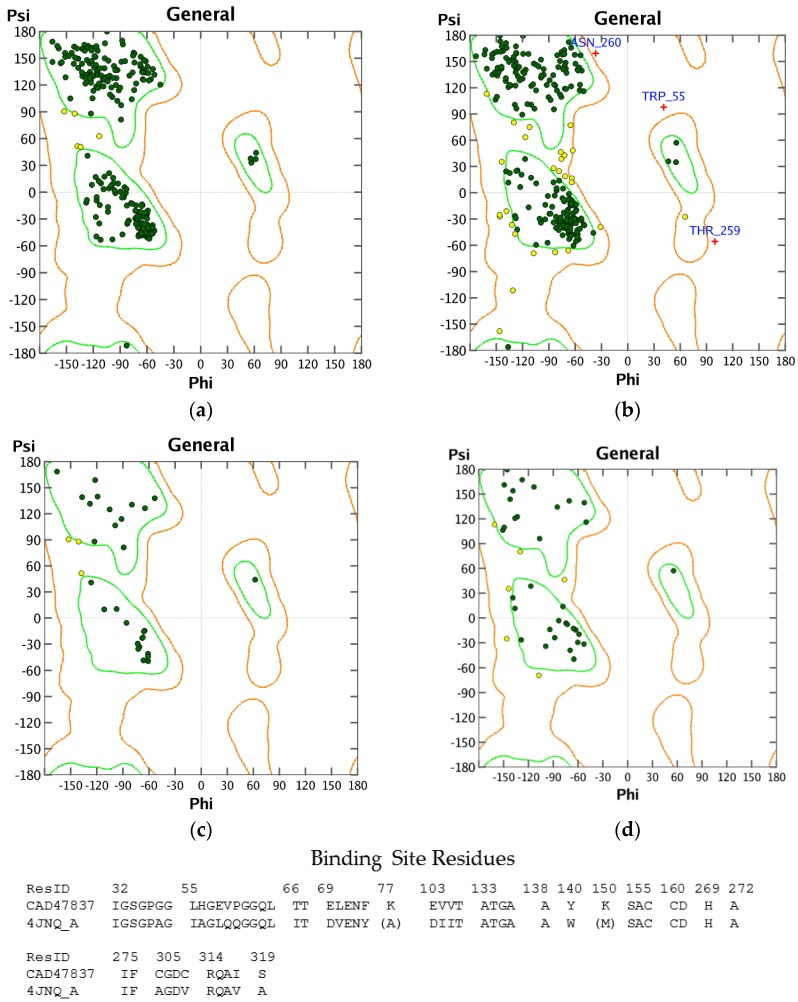
Ramachandran plots of thioredoxin reductase (TrxR) protein structures: (**a**) *Brucella melitensis* TrxR (PDB 4JNQ [24]); (**b**) homology model of *Trichomonas vaginalis* TrxR; (**c**) binding site of BmTrxR; (**d**) binding site of TvTrxR (*T. vaginalis* thioredoxin reductase). ResID: amino acid residue identification.

**Figure 2:**
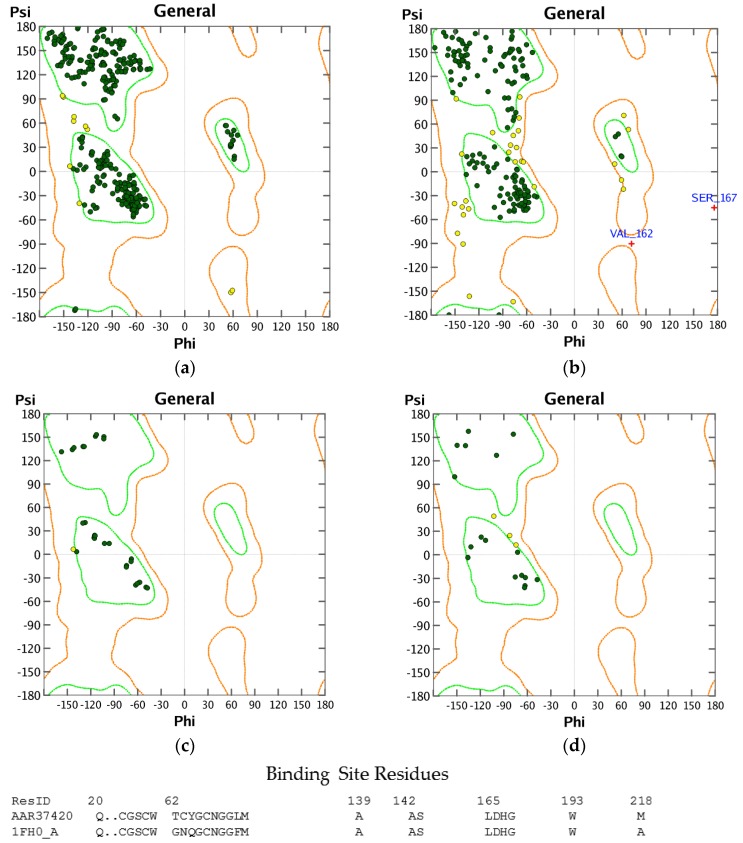
Ramachandran plots of cathepsin V-like protein structures: (**a**) human cathepsin V (HsCatV, PDB 1FH0 [26]); (**b**) homology model of *Trichomonas vaginalis* cathepsin L-like protein, TvCPCAC1; (**c**) binding site of HsCatV; (**d**) binding site of TvCPCAC1.

**Figure 3:**
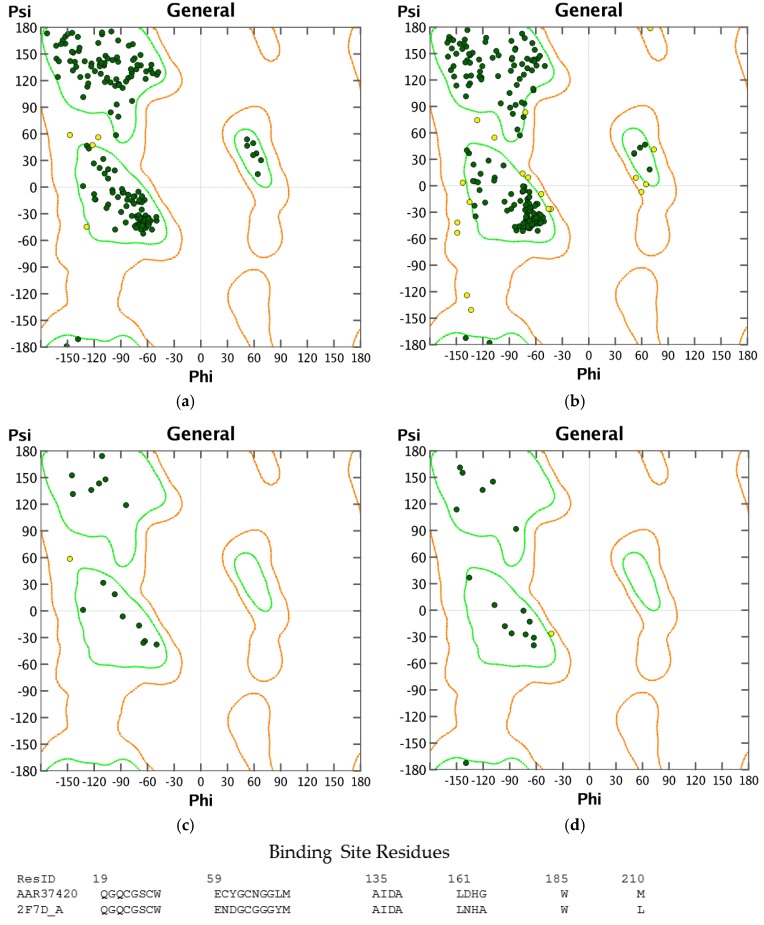
Ramachandran plots of cathepsin K-like protein structures: (**a**) rabbit (*Oryctolagus cuniculus*) cathepsin K (OcCatK, PDB 2F7D [25]); (**b**) homology model of *Trichomonas vaginalis* papain-like protein, TvCP2; (**c**) binding site of OcCatK; (**d**) binding site of TvCP2.

**Figure 4:**
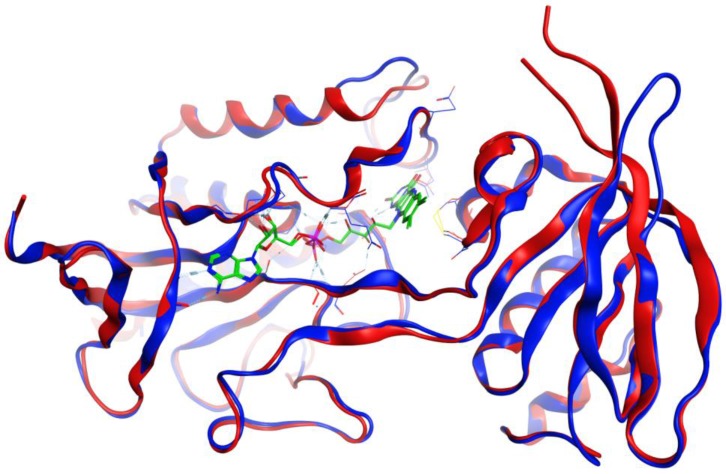
Overlay of the protein structures of *Brucella melitensis* TxR, PDB 4JNQ [24] (red ribbon), and the homology model of *Trichomonas vaginalis* TxR (blue ribbon). The co-crystallized ligand is shown as a wireframe structure.

**Figure 5:**
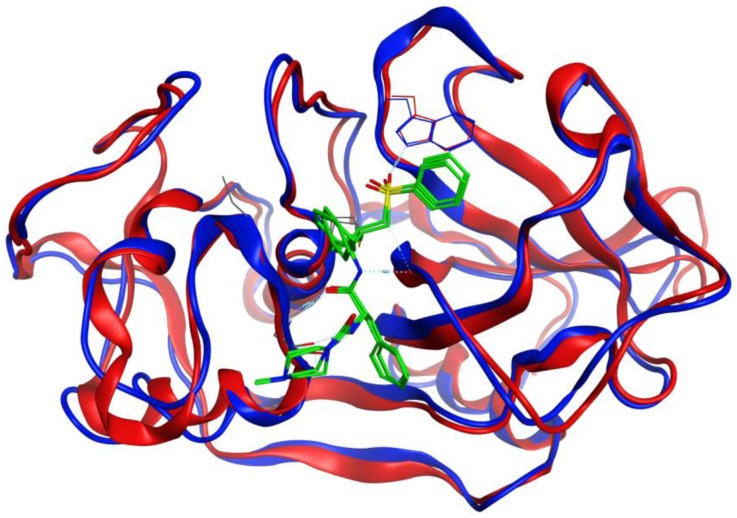
Overlay of the protein structures of human cathepsin V, PDB 1FH0 [26] (red ribbon), and the homology model of *Trichomonas vaginalis* cathepsin L-like protein, TvCPCAC1 (blue ribbon). The co-crystallized ligand is shown as a wireframe structure.

**Figure 6:**
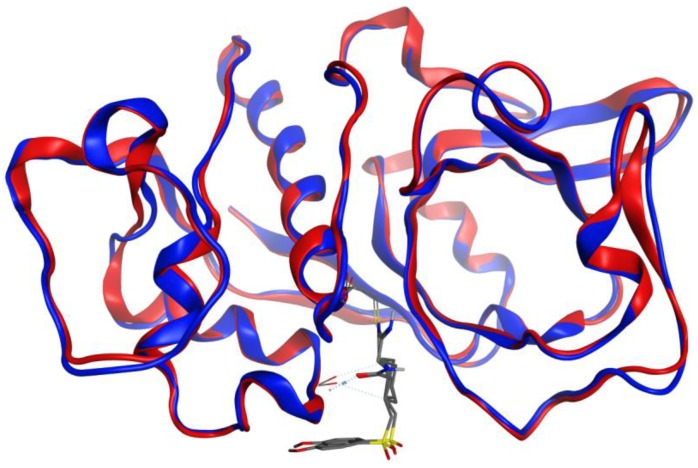
Overlay of the protein structures of rabbit (*Oryctolagus cuniculus*) cathepsin K, PDB 2F7D [25] (red ribbon), and the homology model of *Trichomonas vaginalis* papain-like protein, TvCP2 (blue ribbon). The co-crystallized ligand is shown as a wireframe structure.

Structures in the PDB with co-crystallized ligands in the active sites were chosen as templates for the homology models in order to retain essential binding site topology once the best candidate models were subjected to minimization with the AMBER force field. The Ramachandran plots for the homology models, along with the plots for the template structures are shown in Figure 1, Figure 2 and Figure 3. The Ramachandran plot for modeled *T. vaginalis* thioredoxin reductase (TvTrxR) shows that the phi and psi angles cluster in the typical regions for helices and sheets (Figure 1). As can be seen in the Ramachandran plots of the homology models, outlier residues are not located in the active sites of the target proteins, and the binding site residues lie within the allowed regions of the psi-phi angle islands. Thus, although there are three outliers in the homology model Ramachandran plot of TvTxR, there are none in the active site of this enzyme. As shown in the comparative sequences, the binding site residues are also principally conserved (Figure 1). Likewise, the Ramachandran plots of the homology models of *T. vaginalis* cysteine proteases TvCPCAC1 (Figure 2) and TvCP2 (Figure 3) show the phi and psi angles to cluster in the typical regions for helices and sheets, particularly with the active sites, which showed no residual outliers. In addition, the active sites of the homology modeled protein structures are structurally very similar to the active sites of the proteins from which the models were based (Figure 4, Figure 5 and Figure 6). In the case of the TvTrxR homology model, the RMSD between the crystallized ligand and that of the homology model is 0.703 Å. For the TvCPCAC1 homology model, the RMSD is 0.800 Å. Additionally, in the case of the TvCP2 homology model, the RMSD between the crystallized ligand and that of the homology model is 3.036 Å. Calculated RMSD values include all ligand atoms. Because the models in this study gave reliable backbone conformations, as well as residue interactions around the active sites, these homology models are deemed to be trustworthy, particularly in the regions where molecular docking takes place.

### 3.2. Molecular Docking Validation

**Figure 7:**
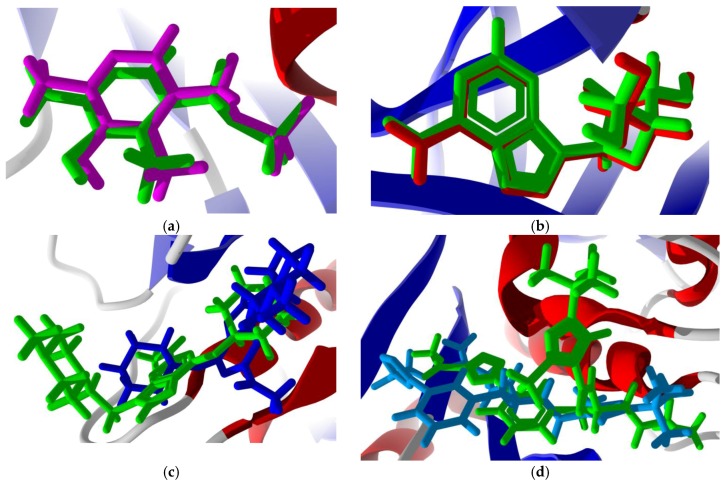
Lowest-energy re-docked poses of co-crystallized ligands: (**a**) *Trichomonas vaginalis* methionine gamma-lyase (TvMGL, PDB 1E5F [27]) showing the co-crystallized ligand, pyridoxal-5′-phosphate (green), and the re-docked ligand (magenta); (**b**) *T. vaginalis* purine nucleoside phosphorylase (TvPNP, PDB 1Z36 [28]) showing the co-crystallized ligand, (1*S*)-1-(7-amino-1*H*-pyrazolo[4,3-d]pyrimidin-3-yl)-1,4-anhydro-d-ribitol (green), and the re-docked ligand (red); (**c**) human cathepsin K (HsCatK, PDB 1U9V [38]) showing the co-crystallized ligand, 6-(cyclohexylamino)-9-[2-(4-methylpiperazin-1-yl)-ethyl]-9*H*-purine-2-carbonitrile (green), and the re-docked ligand (blue); and (**d**) human cathepsin L (HsCatL, PDB 3HWN [39]) showing the co-crystallized ligand, *N*-α-[(3-*t*-Butyl-1-methyl-1*H*-pyrazol-5-yl)carbonyl]-*N*-[(2*E*)-2-iminoethyl]-3-{5-[(*Z*)-iminomethyl]-1,3,4-oxadiazol-2-yl}-l-phenylalaninamide (green), and the re-docked ligand (aqua).

**Figure 8:**
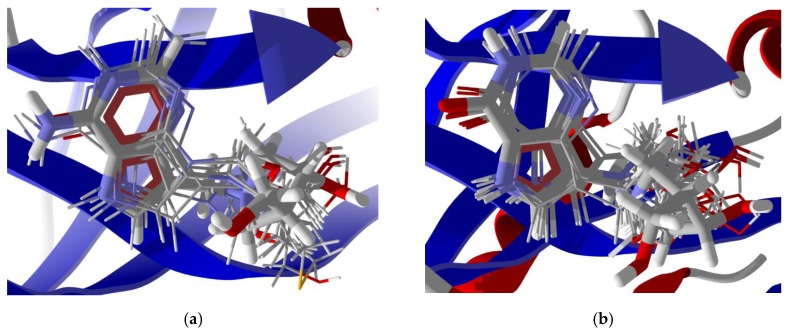
Lowest-energy docked poses of synthetic purine nucleoside phosphorylase inhibitors: (**a**) *Trichomonas vaginalis* purine nucleoside phosphorylase inhibitors [29,53] with TvPNP (PDB: 2ISC [29]; the docked ligands are shown as thin stick figures, while the co-crystallized ligand is shown as a thick stick figure. (**b**) Human purine nucleoside phosphorylase inhibitors [52] with HsPNP (human purine nucleoside phosphorylase) (PDB: 3BGS [33]), the docked ligands are shown as thin stick figures, while the co-crystallized ligand is shown as a thick stick figure.

In order to confirm the validity of the docking method using MolDock, those protein structures with co-crystallized ligands were re-docked to confirm the docking orientation. The docking energies and root mean squared deviations (RMSD, Å) are shown in Table 1. Those structures with relatively rigid co-crystallized ligands reproduced the ligand orientation very well. Thus, for example, TvMGL (PDB 1E5F) and TvPNP (PDB 1Z36) showed excellent re-docking properties with RMSD = 0.57 and 0.21 Å, respectively (Figure 7). On the other hand, co-crystallized ligands with many rotatable bonds were not as successfully re-docked; protein structures with floppy cysteine protease inhibitors, such as HsCatK (PDB 1U9V) and HsCatL (PDB 3HWN), had RMSD values of 4.03 Å and 6.65 Å, respectively (see Figure 7). As an additional validation of the docking method, docking of synthetic picomolar and nanomolar synthetic purine nucleoside phosphorylase inhibitors with human PNP [52] and *T. vaginalis* PNP [53,54] were carried out. The docking of these synthetic inhibitors shows good docking scores, generally <−100 kJ/mol (Table 2) and consistent docking pose orientations (Figure 8) for these compounds compared with the co-crystallized ligands.

### 3.3. Molecular Docking of Phytochemicals

Of the 952 antiprotozoal phytochemicals examined in this molecular docking study, a total of 42 showed notable docking scores (<−125 kJ/mol) (Table 3, Table 4 and Table 5, Figure 9). The −125 kJ cut-off was chosen based on docking scores of co-crystallized ligands and synthetic inhibitors (see Table 1 and Table 2). *T. vaginalis* cysteine proteases do not look to be promising targets for antiprotozoal phytochemicals. None of the phytochemical ligands in this study showed docking energies <−114 kJ/mol (Table 3). Furthermore, most of the ligands showed comparable or better docking to the human cathepsins than to *T. vaginalis* cysteine proteases. Likewise, *T. vaginalis* triosephosphate isomerase does not look to be a promising target for antiprotozoal phytochemicals. Only one ligand, the chalcone 2ʹ,4,4ʹ-trihydroxy-3ʹ,5ʹ-diprenylchalcone, showed promising docking to human triosephosphate isomerase with a normalized docking score of −128.4 kJ/mol (Table 4). There were no phytochemical ligands that showed docking scores to *T. vaginalis* lactate dehydrogenase more exothermic than −116 kJ/mol (Table 4); TvLDH does not look to be a promising target for antiprotozoal phytochemicals. Similarly, the lowest docking score for *T. vaginalis* thioredoxin reductase was −119.8 kJ/mol (Table 4), so TvTrxR cannot be regarded as a potential target for the phytochemicals examined.

**Figure 10:**
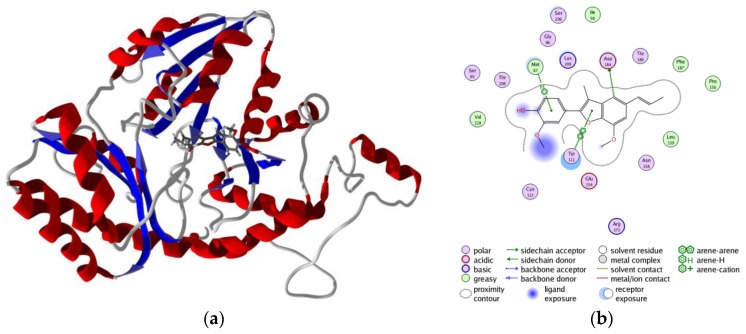
Lowest-energy docked pose of eupomatenoid-7 with *Trichomonas vaginalis* methionine gamma-lyase (TvMGL): (**a**) ribbon diagram of TvMGL with eupomatenoid-7; (**b**) ligand-receptor interaction map of eupomatenoid-7 with TvMGL.

**Figure 11:**
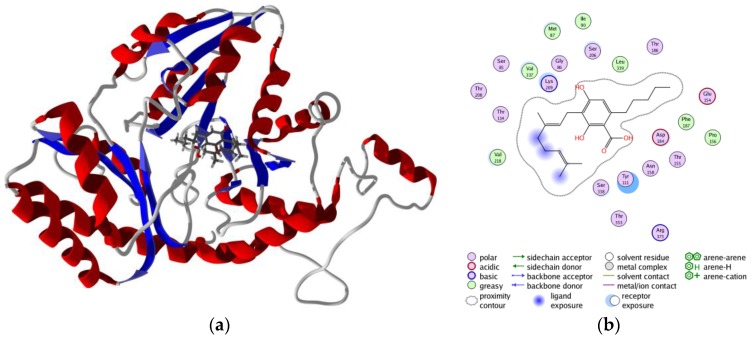
Lowest-energy docked pose of cannabigerolic acid with *Trichomonas vaginalis* methionine gamma-lyase (TvMGL): (**a**) ribbon diagram of TvMGL with cannabigerolic acid; (**b**) ligand-receptor interaction map of cannabigerolic acid with TvMGL.

There were several antiprotozoal phytochemicals that showed potential as *T. vaginalis* methionine gamma-lyase inhibitors with docking scores <−125 kJ/mol (Table 5): the aurone 6-benzoyl-2-[oxomethylpheny]-3-hydroxy-benzofurane (DS_norm_ = −125.5 kJ/mol); the lignans eupomatenoid-5, eupomatenoid-6, and eupomatenoid-7 (DS_norm_ = −130.3, −131.4, and −134.7 kJ/mol, respectively); the cannabinoids 5-acetyl-4-hydroxycannabigerol (DS_norm_ = −127.6 kJ/mol) and cannabigerolic acid (DS_norm_ = −136.2 kJ/mol); and the stilbenoids *trans*-4-(3-methyl-*E*-but-1-enyl)- 3,5,2′,4′-tetrahydroxystilbene (DS_norm_ = −127.8 kJ/mol) and *trans*-4-isopentenyl-3,5,2′,4′- tetrahydroxystilbene (DS_norm_ = −126.8 kJ/mol). The eupomatenoids, particularly eupomatenoid-7, showed promising docking properties. These ligands had docking energies to TvMGL comparable to the co-crystallized ligand, *N*-(hydroxy{3-hydroxy-2-methyl-5-[(phosphonooxy)methyl]pyridin- 4-yl}methyl)norvaline (DS_norm_ = −131.5 kJ/mol). The lowest-energy docking pose of eupomatenoid-7 (Figure 10) showed the ligand in the hydrophobic binding pocket surrounded by Tyr111, Met87, Phe187, Thr186, Asp184 and Ser206. In addition, there was a hydrogen bond interaction between the C(7)-methoxy group of the ligand with the amide −NH_2_ group of Asn158 (Figure 10). Similarly, cannabigerolic acid docked in the same cavity with hydrophobic interactions with Tyr111, Phe187, Thr186 and Asp184, along with hydrogen bonding between the ligand carboxylate and Asn158 and ligand C(2)-O-H with Ser206 (Figure 11).

**Figure 12:**
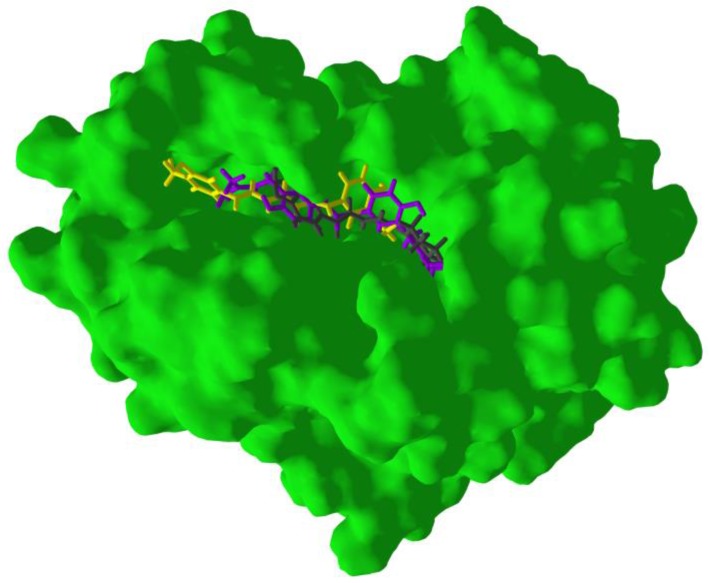
Lowest-energy docked poses of 2′,4,4′-trihydroxy-3,3′-diprenylchalcone (magenta), curcumin (yellow) and piperine (black) with Trichomonas vaginalis purine nucleoside phosphorylase (TvPNP).

**Figure 13:**
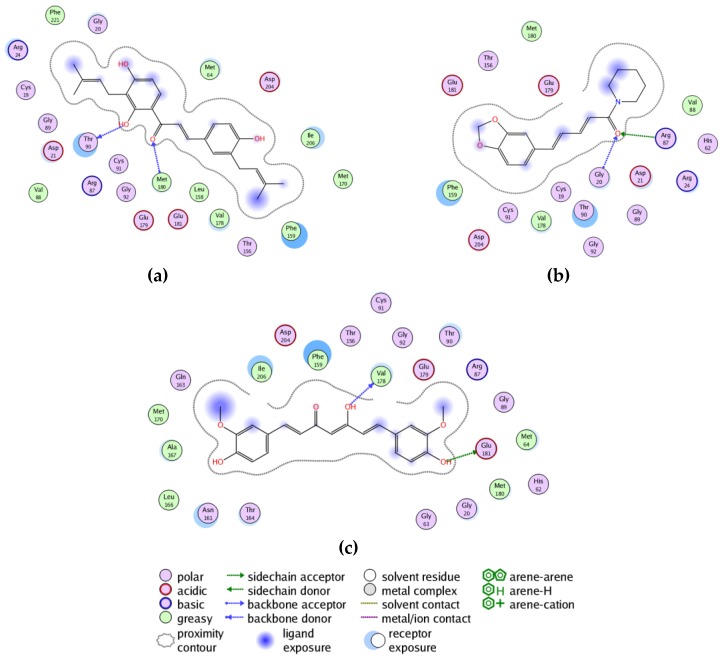
Ligand receptor interaction maps of: (**a**) 2′,4,4ʹ-trihydroxy-3,3ʹ-diprenylchalcone with TvPNP; (**b**) curcumin with TvPNP; and (**c**) piperine with TvPNP.

*T. vaginalis* purine nucleoside phosphorylase is the protein target with the best docking properties to antiprotozoal phytochemicals. There are several phytochemicals that show selective docking to TvPNP over their human homologous isozymes, as well as over the other proteins examined in this study (Table 3). The best docking ligands to TvPNP were polyphenolic phytochemicals, including aurones, chalcones, flavonoids, isoflavonoids and lignans. The overall best docking ligand to TvPNP was 2ʹ,4,4ʹ-trihydroxy-3,3ʹ-diprenylchalcone (DS_norm_ = −141.3 kJ/mol), which docked better to TvPNP than to HsPNP (DS_norm_ = −105.9 kJ/mol). This chalcone also docked more strongly to TvPNP than the co-crystallized ligand (1*S*)-1-(7-amino-1*H*-pyrazolo[4,3-*d*]- pyrimidin-3-yl)-1,4-anhydro-d-ribitol (DS_norm_ = −109.6 kJ/mol) or the synthetic PNP inhibitor SerMe-immucillin (DS_norm_ = −112.7 kJ/mol) [52]. The turmeric constituent curcumin also showed excellent docking to TvPNP (DS_norm_ = −136.1 kJ/mol), but this ligand also docked well with HsPNP (DS_norm_ = −135.3 kJ/mol). The black pepper alkaloid piperine showed selective and strong docking to TvPNP (DS_norm_ = −135.1 kJ/mol). The lowest-energy docked poses of 2ʹ,4,4ʹ-trihydroxy-3,3ʹ-diprenylchalcone, curcumin and piperine are all oriented in a cleft that spans the enzyme active site (Figure 12). Key interactions between these docked ligands and the protein are Thr90, Phe159, Val178, Met180 and Glu179 (Figure 13), and these residues make up the active site of the enzyme.

There are several phytochemicals known to inhibit *T. vaginalis*. These are included in Table 3, Table 4 and Table 5. Although none of the antitrichomonal compounds showed strong docking, the docking analysis does provide some insight into potential protein targets for these compounds. Thus, for example, the germacranolide sesquiterpenoid cnicin, isolated from *Cnicus benedictus*, *Centaurea aspera*, *Centaurea spinosa* and *Centaurea squamosa*, has shown anti-*Trichomonas* activity [42], and this compound showed selective docking to TvPNP (DS_norm_ = −123.6 kJ/mol).

## 4. Conclusions 

Drug resistance of *Trichomonas vaginalis* is increasing, and trichomoniasis can be regarded as a re-emerging infectious disease. There is a need for new chemotherapeutic agents to treat trichomoniasis, and natural products are an attractive resource. Molecular docking of antiprotozoal phytochemical agents has revealed two potential protein drug targets of *T. vaginalis*, methionine gamma-lyase (TvMGL) and purine nucleoside phosphorylase (TvPNP). The best docking ligands were polyphenolic compounds, including aurones, chalcones, flavonoids and lignans. Several phytochemicals showed docking properties superior to co-crystallized ligands or synthetic enzyme inhibitors. This preliminary computational investigation predicts several phytochemicals as potential inhibitors of *T. vaginalis*. However, at this time, we have not provided experimental evidence, in vitro or in vivo, that these predictions will necessarily lead to effective treatments. Additional experimental validation of these predictions is necessary; experimental validation experiments are underway in our laboratories.

## Figures and Tables

**Figure 9:** Phytochemical ligands that showed promising docking properties with *Trichomonas vaginalis* target proteins, as well as known *T. vaginalis* inhibitors and synthetic inhibitors.

**Table:** 

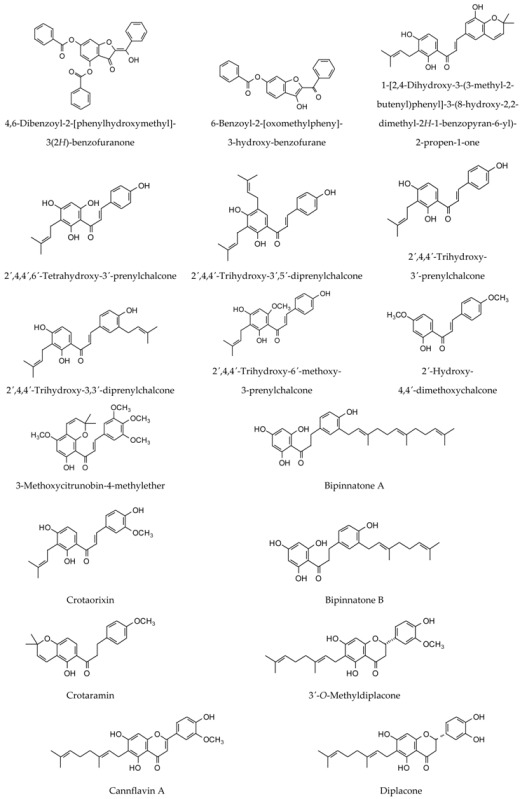		
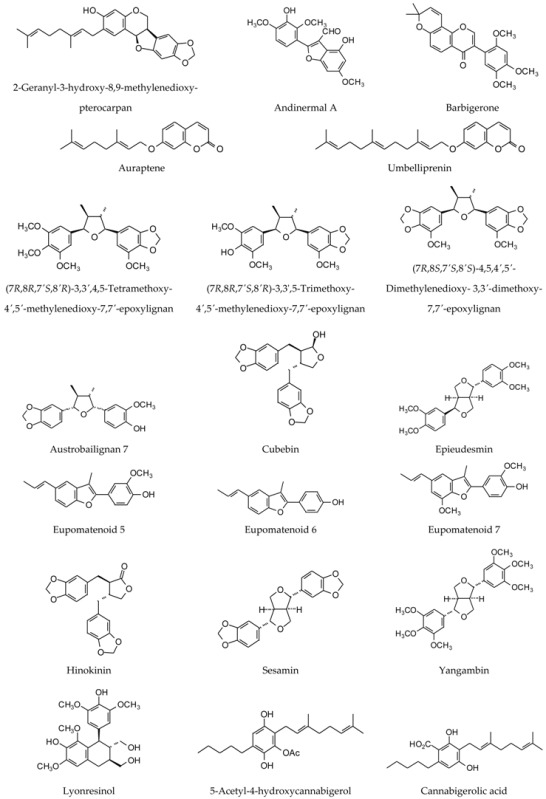		
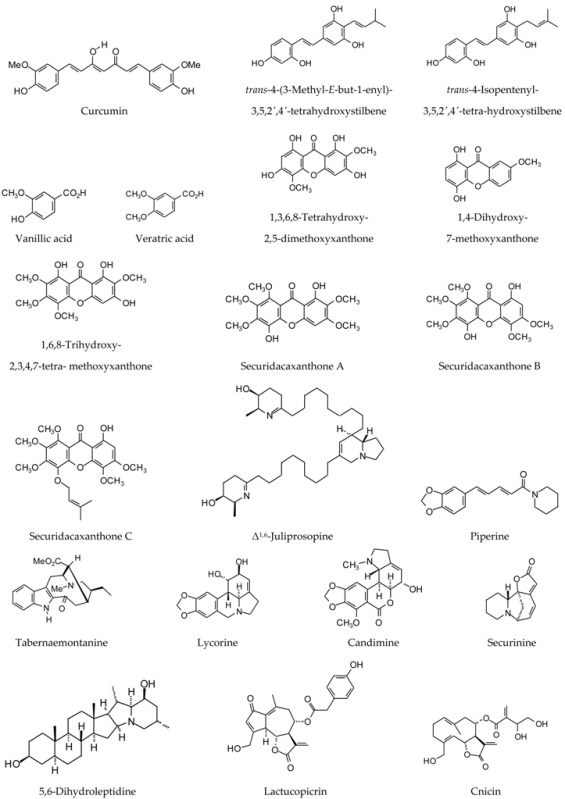		
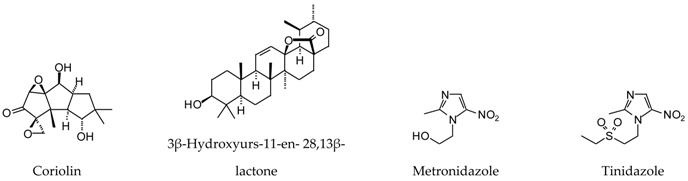		
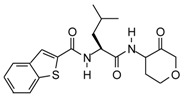 HsCatK inhibitor **43** [54]	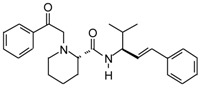 HsCatK inhibitor **44** [54]	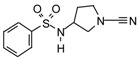 HsCatK inhibitor **45** [54]
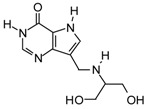 HsPNP inhibitor **4** [52]	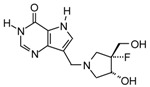 HsPNP inhibitor **5** [52]	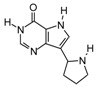 HsPNP inhibitor **12** [52]

**Table 1:** MolDock docking energies of co-crystallized ligands and root mean squared deviations between the co-crystallized ligand and the re-docked poses of the co-crystallized ligand with *Trichomonas vaginalis* and homologous human protein structures.

Protein	PDB code	Co-crystallized ligand	*E*_dock_ (kJ/mol)	RMSD (Å)
TvMGL	1E5E	*N*-(Hydroxy{3-hydroxy-2-methyl-5-[(phosphonooxy)methyl]pyridin-4-yl}methyl)norvaline	-127.6	1.13
	1E5F	Pyridoxal-5′-phosphate	-69.4	0.57
TvCPCAC1	[1FH0] ^a^	*N*-α-[(4-Methylpiperazin-1-yl)carbonyl]-*N*-[(3*S*)-1-phenyl-5-(phenylsulfonyl)pentan-3-yl]-l-phenylalaninamide	-132.6	2.69
TvCP2	[2F7D]	(1*R*,2*R*)-*N*-(2-Aminoethyl)-2-{[(4-methoxyphenyl)sulfonyl]methyl}-cyclohexanecarboxamide	-97.7	1.22
TvPNP	1Z34	2-Fluoro-2′-deoxyadenosine	-97.1	0.91
	1Z36	(1*S*)-1-(7-Amino-1*H*-pyrazolo[4,3-d]pyrimidin-3-yl)-1,4-anhydro-d-ribitol	-97.7	0.21
	2ISC	(3*R*,4*R*)-1-[(4-Amino-5*H*-pyrrolo [3,2-d]pyrimidin-7-yl),ethyl]-4-(hydroxymethyl)pyrrolidin-3-ol	-104.0	0.57
HsCatK	1MEM	*N*-{(1*R*)-3-Phenyl-1-[2-(phenylsulfonyl)ethyl]propyl}-*N*-2-(piperazin-1-ylcarbonyl)-l-leucinamide	-136.2	2.74
	1U9V	6-(Cyclohexylamino)-9-[2-(4-methylpiperazin-1-yl)-ethyl]-9*H*-purine-2-carbonitrile	-41.7	4.03
HsCatL	3HWN	*N*-α-[(3-*t*-Butyl-1-methyl-1*H*-pyrazol-5-yl)carbonyl]-*N*-[(2*E*)-2-iminoethyl]-3-{5-[(*Z*)-iminomethyl]-1,3,4-oxadiazol-2-yl}-l-phenylalaninamide	-135.1	6.65
	3OF8	*N*-α-[(Benzyloxy)carbonyl]-*N*-[(2*S*)-1-(4-*t*-butoxyphenyl)-4-hydroxy-3-oxobutan-2-yl]-l-phenylalaninamide	-120.7	6.43
HsPNP	3BGS	3-Hydroxy-4-hydroxymethyl-1-(4-oxo-4,4a,5,7a-tetrahydro-3*H*-pyrrolo[3,2-*d*]pyrimidin-7-ylmethyl)-pyrrolidinium	-102.8	2.93
	3INY	7-Deazaguanine	-74.0	0.61

^a^ PDB codes in brackets are *Trichomonas vaginalis* homology models based on that protein structure. TvMGL, *T. vaginalis* methionine gamma-lyase; TvCPCAC1, *T. vaginalis* cathepsin L-like protease; TvCP2, *T. vaginalis* papain-like cysteine protease C2; TvPNP, *T. vaginalis* purine nucleoside phosphorylase; HsCatK, human cathepsin K; HsCatL, human cathepsin L; HsPNP, human purine nucleoside phosphorylase.

**Table 2:** MolDock (re-rank) docking scores (E_dock_) and normalized docking scores (DS_norm_) (kJ/mol) for synthetic purine nucleoside phosphorylase inhibitors with *Trichomonas vaginalis* purine nucleoside phosphorylase (TvPNP) and human purine nucleoside phosphorylase (HsPNP).

Synthetic Inhibitor	TvPNP		HsPNP	
	E_dock_	DS_norm_	E_dock_	DS_norm_
HsPNP inhibitor **1** [52]	−102.0	−112.1	−106.7	−117.4
HsPNP inhibitor **2** [52]	−103.9	−116.5	−108.4	−121.6
HsPNP inhibitor **3** [52]	−96.6	−108.0	−103.7	−115.9
HsPNP inhibitor **4** [52]	−96.0	−111.7	−98.8	−114.9
HsPNP inhibitor **5** [52]	−103.9	−114.3	−110.9	−122.0
HsPNP inhibitor **6** [52]	−103.0	−117.8	−105.6	−120.8
HsPNP inhibitor **7** [52]	−102.9	−117.8	−105.5	−120.7
HsPNP inhibitor **8** [52]	−94.6	−107.7	−102.9	−117.1
HsPNP inhibitor **9** [52]	−100.5	−115.1	−108.7	−124.4
HsPNP inhibitor **10** [52]	−101.9	−114.0	−108.5	−121.4
HsPNP inhibitor **11** [52]	−89.3	−106.6	−93.9	−112.1
HsPNP inhibitor **12** [52]	−85.8	−105.1	−95.7	−117.3
HsPNP inhibitor **13** [52]	−86.9	−101.4	−97.7	−114.0
HsPNP inhibitor **14** [52]	−86.4	−100.8	−100.8	−117.7
HsPNP inhibitor **15** [52]	−81.0	−99.2	−88.6	−108.5
HsPNP inhibitor **16** [52]	−93.6	−107.1	−104.2	−119.3
HsPNP inhibitor **17** [52]	−103.9	−116.5	−108.2	−121.3
TvPNP inhibitor **1** [29]	−101.6	−116.4	−98.9	−113.3
TvPNP inhibitor **2** [29]	−102.3	−114.8	−105.3	−118.3
TvPNP inhibitor **3** [29]	−102.9	−117.8	−105.5	−120.8
TvPNP inhibitor **4** [29]	−104.3	−114.8	−118.3	−130.2
TvPNP inhibitor **5** [29]	−103.7	−116.3	−108.4	−121.6
TvPNP inhibitor **6** [29]	−102.6	−115.1	−113.6	−127.6
TvPNP inhibitor **7** [29]	−104.9	−115.7	−107.0	−118.0
TvPNP inhibitor **8** [29]	−102.1	−114.4	−97.2	−109.0
TvPNP inhibitor **9** [29]	−89.7	−98.9	−99.0	−109.2
TvPNP inhibitor formycin A [53]	−101.1	−113.2	−102.2	−114.4

**Table 3:** MolDock (re-rank) docking scores and normalized docking scores (kJ/mol) for *Trichomonas vaginalis* and human cysteine proteases.

Compounds	TvCP2		HsCatK		TvCPCAC1		HsCatL	
	E_dock_	DS_norm_	E_dock_	DS_norm_	E_dock_	DS_norm_	E_dock_	DS_norm_
**AURONES**								
4,6-Dibenzoyl-2-[phenylhydroxymethyl]-3(2*H*)-benzofuranone	−101.2	−93.3	−96.1	−88.6	−103.2	−95.2	−118.8	−109.6
6-Benzoyl-2-[oxomethylpheny]-3-hydroxy-benzofurane	−87.6	−90.3	−92.3	−95.2	−101.9	−105.1	−114.4	−118.0
**CHALCONES**								
1-[2,4-Dihydroxy-3-(3-methyl-2-butenyl)phenyl]-3-(8-hydroxy-2,2-dimethyl-2*H*-1-benzopyran-6-yl)-2-propen-1-one	−68.1	−66.3	−98.0	−95.5	−83.2	−81.0	−113.3	−110.4
2*'*,4,4*'*,6*'*-Tetrahydroxy-3*'*-prenylchalcone	−91.8	−94.8	−94.3	−97.5	−102.0	−105.4	−95.8	−99.0
2*'*,4,4*'*-Trihydroxy-3*'*,5*'*-diprenylchalcone	−82.5	−81.3	−103.0	−101.5	−96.0	−94.6	−112.9	−111.2
2*'*,4,4*'*-Trihydroxy-3*'*-prenylchalcone	−90.5	−95.1	−87.3	−91.7	−99.9	−104.9	−99.8	−104.7
2*'*,4,4*'*-Trihydroxy-3,3*'*-diprenylchalcone	−76.0	−74.8	−98.7	−97.3	−89.1	−87.8	−109.0	−107.4
2*'*,4,4*'*-Trihydroxy-6*'*-methoxy-3-prenylchalcone	−97.8	−99.7	−88.5	−90.3	−102.3	−104.3	−102.7	−104.7
2*'*-Hydroxy-4,4*'*-dimethoxychalcone	−80.3	−88.0	−84.2	−92.4	−86.4	−94.8	−94.6	−103.8
3-Methoxycitrunobin-4-methylether	−92.1	−90.4	−92.4	−90.7	−96.3	−94.6	−110.2	−108.2
Bipinnatone A	−98.8	−91.1	−105.6	−97.4	−103.4	−95.4	−119.9	−110.6
Bipinnatone B	−106.5	−103.4	−109.0	−105.8	−116.5	−113.0	−108.2	−105.1
Crotaorixin	−99.0	−100.9	−92.7	−94.5	−107.6	−109.7	−103.2	−105.2
Crotaramin	−83.0	−85.9	−86.6	−89.7	−91.2	−94.4	−90.5	−93.7
**FLAVONOIDS**								
3*'*-*O*-Methyldiplacone	−97.2	−92.3	−104.3	−99.0	−116.0	−110.2	−105.2	−99.9
Cannflavin A	−100.3	−95.4	−100.3	−95.4	−112.8	−107.3	−113.5	−107.9
Diplacone	−102.7	−98.6	−91.7	−88.0	−117.9	−113.2	−110.3	−105.9
**ISOFLAVONOIDS**								
2-Geranyl-3-hydroxy-8,9-methylenedioxypterocarpan	−89.1	−85.8	−97.3	−93.7	−97.6	−94.0	−113.6	−109.4
Andinermal A	−82.9	−85.3	−86.2	−88.7	−84.0	−86.4	−91.6	−94.3
Barbigerone	−75.1	−73.9	−80.2	−78.9	−86.0	−84.6	−74.8	−73.6
**COUMARINS**								
Auraptene	−83.6	−90.3	−93.1	−100.5	−100.4	−108.4	−103.1	−111.3
Umbelliprenin	−89.1	−89.8	−100.3	−101.1	−93.7	−94.5	−98.2	−98.9
**LIGNANS**								
(7*R*,8*R*,7*'S*,8*'R*)-3,3*'*,4,5-Tetramethoxy-4*'*,5*'*-methylenedioxy-7,7*'*-epoxylignan	−88.4	−85.4	−99.5	−96.1	−107.1	−103.5	−104.6	−101.0
(7*R*,8*R*,7*'S*,8*'R*)-3,3ʹ,5-Trimethoxy-4*'*,5*'*-methylenedioxy-7,7*'*-epoxylignan	−91.4	−89.3	−83.3	−81.4	−94.4	−92.3	−79.9	−78.0
(7*R*,8*S*,7*'S*,8*'S*)-4,5,4*'*,5*'*-Dimethylenedioxy-3,3*'*-dimethoxy-7,7*'*-epoxylignan	−95.7	−93.7	−80.8	−79.1	−98.6	−96.5	−105.0	−102.7
Austrobailignan 7	−86.6	−89.3	−88.7	−91.5	−90.0	−92.8	−89.0	−91.8
Cubebin	−97.0	−98.7	−91.4	−93.0	−96.7	−98.4	−113.4	−115.4
Epieudesmin	−88.3	−87.5	−85.4	−84.6	−95.0	−94.0	−103.9	−102.9
Eupomatenoid 5	−77.9	−84.5	−85.3	−92.5	−93.1	−100.9	−97.9	−106.1
Eupomatenoid 6	−72.9	−81.9	−79.7	−89.6	−86.7	−97.5	−92.6	−104.1
Eupomatenoid 7	−77.0	−80.9	−92.0	−96.6	−98.5	−103.5	−92.8	−97.5
Hinokinin	−99.9	−101.8	−87.6	−89.4	−95.7	−97.6	−109.8	−111.9
Sesamin	−94.9	−96.7	−94.5	−96.3	−99.6	−101.5	−98.1	−100.0
Yangambin	−91.4	−86.3	−75.2	−71.0	−85.6	−80.8	−89.0	−84.0
Lyonresinol ^a^	−84.1	−81.0	−89.3	−86.0	−83.8	−80.7	−76.4	−73.6
**MISCELLANEOUS POLYPHENOLICS**								
5-Acetyl-4-hydroxycannabigerol	−85.4	−85.5	−93.8	−93.9	−91.8	−91.9	−99.0	−99.1
Cannabigerolic acid	−101.1	−102.5	−103.8	−105.2	−109.9	−111.4	−107.6	−109.0
Curcumin	−87.3	−87.8	−99.4	−100.1	−105.3	−106.0	−112.2	−112.9
*trans*-4-(3-Methyl-*E*-but-1-enyl)-3,5,2*'*,4*'*-tetrahydroxystilbene	−85.8	−91.2	−77.3	−82.1	−87.5	−93.1	−107.7	−114.5
*trans*-4-Isopentenyl-3,5,2*'*,4*'*-tetrahydroxystilbene	−74.0	−78.7	−85.5	−90.9	−91.9	−97.7	−101.5	−108.0
Vanillic acid ^a^	−52.5	−68.6	−60.8	−79.4	−57.6	−75.3	−58.0	−75.8
Veratric acid ^a^	−54.1	−68.8	−64.8	−82.4	−59.2	−75.3	−63.2	−80.4
1,3,6,8-Tetrahydroxy-2,5-dimethoxyxanthone ^a^	−70.1	−74.0	−77.5	−81.8	−77.7	−81.9	−79.8	−84.1
1,4-Dihydroxy-7-methoxyxanthone ^a^	−64.4	−72.9	−70.0	−79.2	−74.3	−84.1	−75.7	−85.7
1,6,8-Trihydroxy-2,3,4,7-tetramethoxyxanthone ^a^	−72.6	−73.4	−74.0	−74.8	−71.7	−72.4	−78.5	−79.3
Securidacaxanthone A ^a^	−63.8	−63.6	−72.5	−72.3	−85.3	−85.0	−76.8	−76.6
Securidacaxanthone B ^a^	−63.3	−63.1	−75.7	−75.5	−76.3	−76.1	−72.6	−72.4
Securidacaxanthone C ^a^	−69.3	−65.4	−79.3	−74.9	−48.4	−45.7	−68.5	−64.7
**ALKALOIDS**								
Δ^1,6^-Juliprosopine	−102.6	−86.6	−95.5	−80.6	−104.3	−88.0	−119.6	−100.9
Piperine	−78.2	−85.7	−80.7	−88.4	−90.8	−99.5	−97.9	−107.3
Tabernaemontanine ^a^	−60.7	−61.9	−79.0	−80.6	−62.0	−63.2	−65.0	−66.3
Lycorine ^a^	−71.8	−78.5	−83.9	−91.7	−81.9	−89.6	−75.8	−82.9
Candimine ^a^	−85.4	−87.8	−87.2	−89.7	−77.7	−79.9	−79.3	−81.6
Securinine ^a^	−59.6	−71.5	−59.4	−71.3	−62.4	−74.9	−60.6	−72.7
5,6-Dihydroleptidine ^a^	−45.0	−43.5	−70.7	−68.3	−47.8	−46.2	−91.6	−88.5
**SESQUITERPENOIDS**								
Lactucopicrin	−93.9	−91.2	−93.7	−91.0	−99.2	−96.3	−98.8	−95.9
Cnicin ^a^	−87.2	−87.0	−105.9	−105.6	−96.3	−96.1	−86.9	−86.7
Coriolin ^a^	−68.9	−76.0	−75.5	−83.2	−68.8	−75.9	−77.2	−85.1
**TRITERPENOIDS**								
3β-Hydroxyurs-11-en-28,13β-lactone ^a^	no dock	no dock	−44.1	−41.4	−64.0	−60.1	−56.9	−53.4
**SYNTHETIC INHIBITORS**								
Synthetic cysteine protease inhibitor **43** [54]	−95.2	−94.2	−106.2	−105.0	−69.8	−69.0	−99.9	−98.8
Synthetic cysteine protease inhibitor **44** [54]	−115.1	−112.2	−105.1	−102.6	−110.9	−108.2	−91.7	−89.4
Synthetic cysteine protease inhibitor **45** [54]	−69.8	−79.8	−74.5	−85.1	−73.2	−83.6	−80.4	−91.9

^a^ Natural products known to be *Trichomonas vaginalis* inhibitors.

**Table 4:** MolDock (re-rank) docking scores and normalized docking scores (kJ/mol) for *Trichomonas vaginalis* and human triosephosphate isomerase (TPI), *T. vaginalis* lactate dehydrogenase (LDH) and *T. vaginalis* thioredoxin reductase (TrxR).

Compounds	TvTPI		HsTPI		TvLDH		TvTrxR	
	E_dock_	DS_norm_	E_dock_	DS_norm_	E_dock_	DS_norm_	E_dock_	DS_norm_
**AURONES**								
4,6-Dibenzoyl-2-[phenylhydroxymethyl]-3(2*H*)-benzofuranone	−106.0	−97.8	−118.3	−109.1	−112.0	−103.3	−113.5	−104.7
6-Benzoyl-2-[oxomethylpheny]-3-hydroxy-benzofurane	−99.4	−102.5	−97.9	−101.0	−103.9	−107.1	−105.1	−108.4
**CHALCONES**								
1-[2,4-Dihydroxy-3-(3-methyl-2-butenyl)phenyl]-3-(8-hydroxy-2,2-dimethyl-2*H*-1-benzopyran-6-yl)-2-propen-1-one	−102.7	−100.1	−119.1	−116.0	−99.5	−96.9	−113.8	−110.9
2*′*,4,4*′*,6*′*-Tetrahydroxy-3*′*-prenylchalcone	−100.7	−104.1	−105.3	−108.8	−96.7	−99.9	−75.1	−77.6
2*′*,4,4*′*-Trihydroxy-3*′*,5*′*-diprenylchalcone	−112.9	−111.3	−130.3	−128.4	−102.9	−101.4	−93.3	−91.9
2*′*,4,4*′*-Trihydroxy-3*′*-prenylchalcone	−99.9	−104.9	−105.4	−110.6	−95.1	−99.9	−101.7	−106.8
2*′*,4,4*′*-Trihydroxy-3,3*′*-diprenylchalcone	−106.2	−104.6	−102.7	−101.2	−103.1	−101.6	−89.5	−88.2
2*′*,4,4*′*-Trihydroxy-6*′*-methoxy-3-prenylchalcone	−102.9	−104.9	−113.3	−115.5	−95.6	−97.5	−95.8	−97.6
2*′*-Hydroxy-4,4*′*-dimethoxychalcone	−86.9	−95.4	−91.1	−100.0	−87.7	−96.2	−85.8	−94.1
3-Methoxycitrunobin-4-methylether	−101.6	−99.8	−97.5	−95.8	−96.7	−95.0	−89.2	−87.6
Bipinnatone A	−104.6	−96.4	−107.0	−98.7	−105.4	−97.2	−113.2	−104.4
Bipinnatone B	−108.9	−105.7	−124.2	−120.6	−113.9	−110.6	−123.5	−119.8
Crotaorixin	−96.9	−98.7	−106.9	−109.0	−100.0	−101.9	−105.7	−107.8
Crotaramin	−95.7	−99.1	−101.0	−104.6	−89.2	−92.4	−83.7	−86.6
**FLAVONOIDS**								
3*′*-*O*-Methyldiplacone	−98.0	−93.0	−100.3	−95.2	−112.5	−106.9	−92.1	−87.5
Cannflavin A	−98.6	−93.8	−107.8	−102.5	−104.8	−99.7	−112.6	−107.0
Diplacone	−97.3	−93.4	−109.8	−105.4	−101.3	−97.2	−102.2	−98.1
**ISOFLAVONOIDS**								
2-Geranyl-3-hydroxy-8,9-methylenedioxypterocarpan	−95.5	−92.0	−110.5	−106.4	−119.5	−115.1	−112.6	−108.4
Andinermal A	−92.4	−95.1	−88.8	−91.4	−86.4	−89.0	−102.2	−105.2
Barbigerone	−81.4	−80.0	−86.8	−85.4	−87.3	−85.9	−66.3	−65.3
**COUMARINS**								
Auraptene	−90.5	−97.7	−93.4	−100.8	−88.6	−95.7	−94.5	−102.0
Umbelliprenin	−93.0	−93.7	−113.0	−113.9	−95.8	−96.6	−90.4	−91.1
**LIGNANS**								
(7*R*,8*R*,7*'S*,8*'R*)-3,3*'*,4,5-Tetramethoxy-4*'*,5*'*-methylenedioxy-7,7*'*-epoxylignan	−91.7	−88.6	−97.5	−94.2	−105.2	−101.6	−85.5	−82.6
(7*R*,8*R*,7*'S*,8*'R*)-3,3*′*,5-Trimethoxy-4*′*,5*′*-methylenedioxy-7,7*′*-epoxylignan	−94.7	−92.5	−92.8	−90.6	−102.3	−100.0	−59.7	−58.3
(7*R*,8*S*,7*′S*,8*′S*)-4,5,4*′*,5*′*-Dimethylenedioxy-3,3*′*-dimethoxy-7,7*′*-epoxylignan	−100.2	−98.0	−107.7	−105.4	−102.6	−100.4	−91.1	−89.2
Austrobailignan 7	−95.3	−98.2	−93.8	−96.7	−96.4	−99.4	−95.8	−98.8
Cubebin	−98.9	−100.6	−104.9	−106.7	−98.0	−99.8	−62.6	−63.7
Epieudesmin	−96.3	−95.4	−89.6	−88.7	−91.7	−90.8	−85.2	−84.4
Eupomatenoid 5	−91.0	−98.7	−88.5	−96.0	−85.6	−92.9	−92.0	−99.8
Eupomatenoid 6	−80.5	−90.5	−88.7	−99.7	−79.9	−89.8	−90.8	−102.1
Eupomatenoid 7	−87.9	−92.2	−95.7	−100.5	−93.6	−98.3	−95.9	−100.6
Hinokinin	−103.0	−105.1	−96.3	−98.2	−98.5	−100.5	−78.5	−80.0
Sesamin	−93.8	−95.6	−101.8	−103.7	−93.6	−95.5	−90.1	−91.8
Yangambin	−100.6	−95.0	−95.5	−90.2	−93.3	−88.1	−64.7	−61.1
Lyonresinol ^a^	−104.7	−100.9	−101.1	−97.3	−100.3	−96.6	−92.6	−89.1
**MISCELLANEOUS POLYPHENOLICS**								
5-Acetyl-4-hydroxycannabigerol	−88.0	−88.1	−98.1	−98.2	−93.2	−93.3	−105.3	−105.4
Cannabigerolic acid	−92.3	−93.6	−99.2	−100.6	−93.8	−95.1	−113.0	−114.6
Curcumin	−101.4	−102.0	−108.7	−109.4	−101.5	−102.2	−115.6	−116.3
*trans*-4-(3-Methyl-*E*-but-1-enyl)-3,5,2*'*,4*'*-tetrahydroxystilbene	−82.1	−87.3	−106.0	−112.7	−90.1	−95.8	−104.6	−111.3
*trans*-4-Isopentenyl-3,5,2*'*,4*'*-tetrahydroxystilbene	−89.2	−94.8	−103.3	−109.8	−84.5	−89.8	−99.0	−105.3
Vanillic acid ^a^	−63.1	−82.5	−61.7	−80.6	−61.0	−79.8	−64.3	−84.1
Veratric acid ^a^	−66.4	−84.5	−66.6	−84.7	−64.5	−82.1	−68.4	−87.1
1,3,6,8-Tetrahydroxy-2,5-dimethoxyxanthone ^a^	−85.8	−90.5	−71.5	−75.4	−75.9	−80.1	−75.2	−79.3
1,4-Dihydroxy-7-methoxyxanthone ^a^	−76.0	−86.1	−66.6	−75.5	−72.8	−82.5	−64.8	−73.4
1,6,8-Trihydroxy-2,3,4,7-tetramethoxyxanthone ^a^	−85.2	−86.1	−65.4	−66.0	−78.5	−79.3	−73.8	−74.5
Securidacaxanthone A ^a^	−88.3	−88.0	−76.7	−76.5	−76.3	−76.1	−71.8	−71.6
Securidacaxanthone B ^a^	−85.0	−84.8	−74.4	−74.2	−74.9	−74.7	−70.3	−70.2
Securidacaxanthone C ^a^	−78.9	−74.5	−81.3	−76.8	−82.3	−77.7	−78.5	−74.1
**ALKALOIDS**								
Δ^1,6^-Juliprosopine	−107.2	−90.5	−113.2	−95.5	−112.7	−95.1	−97.9	−82.5
Piperine	−82.3	−90.1	−87.1	−95.4	−83.1	−91.1	−77.3	−84.7
Tabernaemontanine ^a^	−76.6	−78.1	−75.6	−77.1	−83.3	−84.9	−75.4	−76.8
Lycorine ^a^	−71.8	−78.4	−81.0	−88.5	−77.9	−85.1	−74.5	−81.5
Candimine ^a^	−86.0	−88.4	−77.6	−79.7	−86.6	−89.1	−72.6	−74.7
Securinine ^a^	−65.1	−78.1	−60.5	−72.5	−70.7	−84.8	−54.0	−64.7
5,6-Dihydroleptidine ^a^	−54.2	−52.3	−77.7	−75.1	−91.4	−88.4	−56.6	−54.7
**SESQUITERPENOIDS**								
Lactucopicrin	−105.8	−102.7	−99.8	−96.9	−102.5	−99.5	−99.6	−96.7
Cnicin ^a^	−99.6	−99.4	−102.7	−102.4	−98.0	−97.7	−92.5	−92.3
Coriolin ^a^	−73.2	−80.7	−78.6	−86.7	−72.4	−79.7	−71.7	−79.0
**TRITERPENOIDS**								
3β-Hydroxyurs-11-en-28,13β-lactone ^a^	−47.7	−44.8	−63.7	−59.7	−70.0	−65.7	−50.7	−47.6
**SYNTHETIC INHIBITORS**								
Metronidazole							−64.4	−83.7
Tinidazole							−78.5	−90.2

^a^ Natural products known to be *Trichomonas vaginalis* inhibitors.

**Table 5:** MolDock (re-rank) docking scores and normalized docking scores (kJ/mol) for *Trichomonas vaginalis* methionine gamma-lyase (MGL) and *T. vaginalis* and human purine nucleoside phosphorylase (PNP).

Compounds	TvMGL		TvPNP		HsPNP	
	E_dock_	DS_norm_	E_dock_	DS_norm_	E_dock_	DS_norm_
**AURONES**						
4,6-Dibenzoyl-2-[phenylhydroxymethyl]-3(2*H*)-benzofuranone	−98.8	−91.2	−141.6	−130.6	−118.3	−109.1
6-Benzoyl-2-[oxomethylpheny]-3-hydroxy-benzofurane	−121.7	−125.5	−130.8	−134.9	−120.2	−124.0
**CHALCONES**						
1-[2,4-Dihydroxy-3-(3-methyl-2-butenyl)phenyl]-3-(8-hydroxy-2,2-dimethyl-2*H*-1-benzopyran-6-yl)-2-propen-1-one	−118.5	−115.4	−137.2	−133.6	−92.1	−89.7
2*'*,4,4*'*,6*'*-Tetrahydroxy-3*'*-prenylchalcone	−115.7	−119.6	−122.2	−126.3	−112.3	−116.1
2*'*,4,4*'*-Trihydroxy-3*'*,5*'*-diprenylchalcone	−97.4	−96.0	−134.9	−132.9	−119.1	−117.3
2*'*,4,4*'*-Trihydroxy-3*'*-prenylchalcone	−116.0	−121.8	−122.9	−129.0	−111.6	−117.2
2*'*,4,4*'*-Trihydroxy-3,3*'*-diprenylchalcone	−126.5	−124.7	−143.4	−141.3	−107.5	−105.9
2*'*,4,4*'*-Trihydroxy-6*'*-methoxy-3-prenylchalcone	−107.1	−109.2	−130.6	−133.2	−124.2	−126.6
2*'*-Hydroxy-4,4*'*-dimethoxychalcone	−103.5	−113.6	−114.9	−126.0	−100.7	−110.5
3-Methoxycitrunobin-4-methylether	−102.5	−100.6	−129.5	−127.1	−108.7	−106.8
Bipinnatone A	−122.7	−113.1	−146.4	−135.1	−142.5	−131.4
Bipinnatone B	−120.3	−116.7	−139.3	−135.2	−133.4	−129.5
Crotaorixin	−120.2	−122.5	−127.3	−129.7	−117.0	−119.3
Crotaramin	−114.8	−118.9	−121.1	−125.3	−107.1	−110.9
**FLAVONOIDS**						
3*'*-*O*-Methyldiplacone	−106.2	−100.8	−135.6	−128.8	−117.3	−111.4
Cannflavin A	−118.0	−112.2	−138.5	−131.7	−125.4	−119.3
Diplacone	−109.2	−104.8	−137.5	−132.0	−133.7	−128.4
**ISOFLAVONOIDS**						
2-Geranyl-3-hydroxy-8,9-methylenedioxypterocarpan	−75.5	−72.7	−139.1	−134.0	−125.9	−121.3
Andinermal A	−104.6	−107.7	−121.4	−125.0	−104.0	−107.0
Barbigerone	−94.9	−93.4	−132.3	−130.1	−100.2	−98.6
**COUMARINS**						
Auraptene	−104.5	−112.9	−117.0	−126.3	−107.3	−115.8
Umbelliprenin	−119.3	−120.3	−129.9	−130.9	−110.3	−111.2
**LIGNANS**						
(7*R*,8*R*,7*'S*,8*'R*)-3,3*'*,4,5-Tetramethoxy-4*'*,5*'*-methylenedioxy-7,7*'*-epoxylignan	−76.2	−73.6	−131.8	−127.4	−99.0	−95.6
(7*R*,8*R*,7*'S*,8*'R*)-3,3ʹ,5-Trimethoxy-4*'*,5*'*-methylenedioxy-7,7*'*-epoxylignan	−89.7	−87.6	−129.7	−126.7	−107.6	−105.1
(7*R*,8*S*,7*'S*,8*'S*)-4,5,4*'*,5*'*-Dimethylenedioxy-3,3*'*-dimethoxy-7,7*'*-epoxylignan	−95.7	−93.7	−134.2	−131.3	−101.2	−99.1
Austrobailignan 7	−85.0	−87.6	−122.9	−126.8	−106.1	−109.4
Cubebin	−110.0	−112.0	−125.9	−128.1	−110.2	−112.1
Epieudesmin	−94.2	−93.3	−129.9	−128.7	−100.8	−99.9
Eupomatenoid 5	−120.1	−130.3	−110.7	−120.1	−121.1	−131.3
Eupomatenoid 6	−116.9	−131.4	−105.7	−118.8	−113.1	−127.1
Eupomatenoid 7	−128.3	−134.7	−112.0	−117.6	−111.7	−117.3
Hinokinin	−113.2	−115.4	−124.5	−126.9	−105.6	−107.6
Sesamin	−104.4	−106.4	−127.3	−129.8	−106.3	−108.4
Yangambin	−97.0	−91.6	−134.1	−126.6	−97.8	−92.3
Lyonresinol ^a^	−50.0	−48.1	−99.7	−96.0	−91.0	−87.6
**MISCELLANEOUS POLYPHENOLICS**						
5-Acetyl-4-hydroxycannabigerol	−127.4	−127.6	−115.6	−115.7	−106.0	−106.1
Cannabigerolic acid	−134.3	−136.2	−122.5	−124.1	−112.4	−113.9
Curcumin	−113.9	−114.6	−135.2	−136.1	−134.5	−135.3
*trans*-4-(3-Methyl-*E*-but-1-enyl)-3,5,2*'*,4*'*-tetrahydroxystilbene	−120.2	−127.8	−117.8	−125.3	−117.5	−124.9
*trans*-4-Isopentenyl-3,5,2*'*,4*'*-tetrahydroxystilbene	−119.2	−126.8	−115.3	−122.6	−111.3	−118.3
Vanillic acid ^a^	−72.5	−94.7	−70.9	−92.6	−70.1	−91.7
Veratric acid ^a^	−75.7	−96.3	−70.9	−90.2	−72.0	−91.6
1,3,6,8-Tetrahydroxy-2,5-dimethoxyxanthone ^a^	−93.2	−98.2	−91.3	−96.3	−91.2	−96.2
1,4-Dihydroxy-7-methoxyxanthone ^a^	−85.2	−96.5	−86.8	−98.3	−99.3	−112.5
1,6,8-Trihydroxy-2,3,4,7-tetramethoxyxanthone ^a^	−95.8	−96.8	−92.9	−93.9	−85.3	−86.2
Securidacaxanthone A ^a^	−78.1	−77.9	−92.2	−92.0	−60.1	−60.0
Securidacaxanthone B ^a^	−91.8	−91.5	−93.9	−93.7	−69.1	−68.9
Securidacaxanthone C ^a^	−104.5	−98.6	−108.6	−102.5	−82.6	−78.0
**ALKALOIDS**						
Δ^1,6^-Juliprosopine	−113.8	−96.0	−159.7	−134.7	−117.8	−99.3
Piperine	−79.8	−87.5	−123.3	−135.1	−101.1	−110.8
Tabernaemontanine ^a^	−55.0	−56.0	−91.3	−93.1	−88.0	−89.7
Lycorine ^a^	−85.3	−93.3	−88.1	−96.3	−88.8	−97.1
Candimine ^a^	−72.7	−74.8	−89.5	−92.0	−89.4	−91.9
Securinine ^a^	−66.7	−80.0	−75.1	−90.1	−80.0	−96.0
5,6-Dihydroleptidine ^a^	−27.4	−26.4	−86.0	−83.2	−90.2	−87.2
**SESQUITERPENOIDS**						
Lactucopicrin	−115.0	−111.6	−133.1	−129.2	−125.4	−121.8
Cnicin ^a^	−69.8	−69.6	−123.9	−123.6	−97.0	−96.7
Coriolin ^a^	no dock	no dock	−87.9	−96.9	−83.0	−91.5
**TRITERPENOIDS**						
3β-Hydroxyurs-11-en-28,13β-lactone ^a^	−15.2	−14.3	−69.9	−65.6	−73.2	−68.7
**SYNTHETIC INHIBITORS**						
Purine nucleoside phosphorylase inhibitor **4** [52]	−101.4	−118.0	−96.9	−112.7	−102.6	−119.3
Purine nucleoside phosphorylase inhibitor **5** [52]	−94.3	−103.7	−103.9	−114.3	−111.5	−122.6
Purine nucleoside phosphorylase inhibitor **12** [52]	−84.4	−103.4	−85.8	−105.1	−95.7	−117.2

^a^ Natural products known to be *Trichomonas vaginalis* inhibitors.
